# Risk Communication and Community Engagement in Action During Ukraine’s War

**DOI:** 10.5334/aogh.3937

**Published:** 2022-11-16

**Authors:** Diana Maddah, Cristiana Salvi, Ramnath Vadi, Mariam Mohammad

**Affiliations:** 1 UK-Med- Health Department, UK; 2 American University of Beirut, Faculty of Health Sciences, LB; 3 WHO, Regional Office for Europe, Copenhagen, DK

**Keywords:** Risk Communication and Community Engagement, Participatory Approach, War, Refugees

## Abstract

In the light of recent emergencies in Europe and around the globe—including COVID-19 and the war in Ukraine—the spotlight has shifted towards the scarcity of Risk Communication and Community Engagement (RCCE) research applied to health emergencies. RCCE nurtures the sense of empowerment among communities since it ensures that individuals and communities are part of the solution creation, thus they take informed decisions to protect their health and in turn, contribute to emergency control. Therefore, RCCE can play an important role as core public health intervention across health emergency preparedness and response. However, its tremendous impact, is still underestimated and not widely common. This viewpoint showcases the RCCE measures applied to the Ukrainian emergency to ensure that Ukrainian refugees access health services in host countries, based on their needs and concerns.

## Background

The essence of RCCE is working together with the community in order to co-design behavioural, social and public health solutions that are tailored to their needs and adapted to the evolution of the emergency and risk perceptions over time [1]. Consequently, there is an increase in adherence to recommended preventive measures since they are created with the people, and for the people. This increase contributes to reducing exposure, suppressing transmission, protecting the vulnerable, and reducing deaths as an end point [1].

Trust is a central factor to RCCE, as a trustworthy relationship between responders and communities is at the basis of public support to health emergencies’ response. In preparedness, RCCE strengthens the capacities and capabilities of countries to communicate risks to specific population groups based on their perceptions and concerns and by engaging trusted community actors [2]. Moreover, in emergency response, RCCE builds trust, helps prevent and manage infodemics by providing individuals and communities with actionable, timely, and credible health information online and offline [2]. Hence, it is critical that RCCE is sustainably resourced and integrated into national preparedness plans [2].

In the context of the war in Ukraine, the WHO Regional Office for Europe attributed a critical role to RCCE within the response plan in both Ukraine and surrounding countries. To roll out a strong RCCE response in refugee-receiving countries, the Communication Pillar of the Ukraine’s emergency Incident Management Support Team (IMST) prioritized strengthening RCCE capacities in the WHO country offices of the Czech Republic, Hungary, Poland, the Republic of Moldova, Romania, and Slovakia to ensure adequate support to respective countries [3].

## RCCE Plan: From Assessment till Recommended Interventions

### Rapid situational analysis

A RCCE rapid needs assessment was conducted across the countries in the initial weeks of the war, to understand the health needs, perceptions and concerns reported by Ukrainian refugees, along with capacity of each country to respond to these needs, while maintaining social cohesion. Interview guides were designed to interview civil society organizations (CSOs), governmental figures (ministries and municipalities), key leaders, refugees, and host communities. These interview guides included the following themes: Scope of work (geographical area, challenges faced, targeted group, support needed); coordination mechanism with other partners; Socio-demographics of the refugees; RCCE (Knowledge: level of health literacy and any health related knowledge gap; Practices: ways of communications between refugees and CSOs, language used, tools/materials used; and Attitude: perceptions of refugees, fear and concerns, any cultural sensitivity); Communication channels (Offline/online social media, word of mouth, friends, door to door activities, mass media, and influencers identification (trustworthy leaders). Finally, the last theme is related to the misconceptions/rumours the refugees receive as well as the perception of the host communities towards them. Fifteen focus groups discussions and 71 interviews were held with the different stakeholders with different surrounding European countries. This situational analysis led to objectives prioritization.

The notes taken through the focus group discussions and in-depth interviews were analysed using a rapid analysis process to identify key themes and inform the development of interventions in a timely manner [4].

### Overall findings

The majority of the Ukrainian refugees were women (45%), men (12%) and children under 18 (43%). Although there were some discrepancies in the findings between different countries, in this report we highlighted some common challenges. The majority of persons arriving from Ukraine were Ukrainian citizens. As reported by CSOs and refugees the following health issues were prominent:

- Upper respiratory infections due to low temperature and long trips to arrive in the host countries.
- Exhaustion.
- Psychosomatic stress reactions (headache, shaking).
- Gastro-intestinal conditions (diarrhoea, vomit).
- Heart conditions (hypertension, arrhythmia).
- Chronic conditions (diabetes, cancer, disabilities).

As for the access to healthcare system, all holders of the special long-term visa (valid for a year) could register and get health insurance free of charge in the host countries. Regardless, many Ukrainian refugees still preferred to rely on their Ukrainian physicians, whether online or in-person. Prescription medications and vaccinations were provided for free. However, many Ukrainians had the fear to change the medications they used to take in Ukraine to new ones in the host countries as once they went back, they’d have to change medicines again. As for the vaccination, if the child’s parents or other persons do not provide documentation of his/her vaccination, he or she will be treated as unvaccinated, and vaccination will be given to the child. However, many Ukrainian refugees are not ready to vaccinate their children. More hesitancy was noticed regarding COVID-19 vaccines uptakes related to concerns for side effects and toxicity of the vaccines. When it comes to psychosocial support, refugees especially children were open to it. But limited available number of licensed psychologists and psychotherapists posed a challenge to address this need. Some CSOs provided mental health support by integrating Ukrainian social workers as volunteers to support; However, more capacity building is needed to ensure the quality and sustainability of the services.

All the ministries of health in the host countries posted health information on their governmental websites. The refugees were not always aware of these efforts; thus, accessing health information is a challenge for many. The information channels that generally Ukrainian refugees prefer to use were both online and offline, with a preference for the online channels for youth and offline channels for older people. Refugees show a high sense of gratitude towards the hosting system and population.

As for the host communities’ perceptions, there was an initial overwhelming welcome and solidarity from the resident population. There is a very considerable number of Ukrainian refugees who have relatives and friends in the European countries who hosted them. Some cases of frustration towards refugees by the host communities have been already observed due to the perceived competition for resources (mainly schools and health care services).

Based on the above findings, the goal across all the countries was to support Ministries of Health’s Risk Communication and Community Engagement efforts to encourage Ukrainian refugees to access health services, based on their needs and concerns. Four main objectives were identified:

- **Objective 1:** Strengthen trust between refugees, host population and health authorities.
- **Objective 2:** Support refugees to access health services.
- **Objective 3:** Promote healthy behaviours among refugees and host population.
- **Objective 4:** Strengthen community readiness and resilience.

### Strategic Plan Pillars

**Figure 1:**
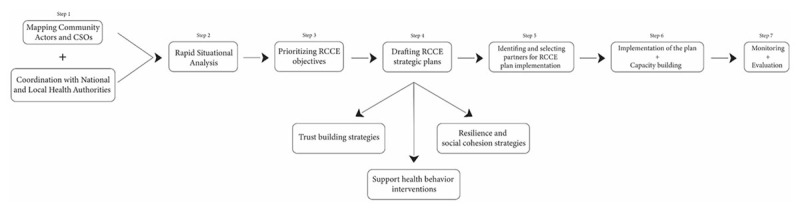
Steps of the RCCE Plan.

All the RCCE plans covering the health sector’s response to the refugees’ emergency are part of the overall inter-sectoral response plan Figure 1. They rely on three main success factors:

*1. Drivers related to behaviour changes*.

Understanding both individual behavioural variables and the social and societal systems that underpin the behaviour is necessary to making health care available, acceptable, convenient, and appealing to individuals [5]. According to research, people are more likely to trust organisations when they believe that their actions are characterised by consistency, competence, justice, objectivity, empathy, or sincerity [6]. People may make wise decisions, protect themselves, and follow advised practises when such measures are also simple to understand [7,8]. In a situation of extreme confusion and anxiety as that of Ukrainian refugees fleeing the war, receiving clear information is vital. This will improve refugees’ ability to navigate and access the new system for prevention and treatment, as well as give them a sense of orientation and belonging, thus increasing their well-being. Theoretical crisis models recommend that it is essential to comprehend public views; yet, little is understood about the intricate interactions between health seeking behaviour and the numerous elements that influence persons escaping war [9,10].

*2. Human interactions at the core of behavioural change*.

Understanding people’s perceptions; social and physical situations, and psychological requirements is essential for suitable and effective interventions [11]. RCCE interventions need to leverage human relations and interactions at most. While Information, Education, Communication (IEC) materials are a useful support tool, it is the direct engagement with affected communities and stakeholders that can make a difference in terms of behavioural change and perceived support. The role of local authorities, civil society organizations, and community workers is critical to this objective. They have the outreach, the trust, and the capacity to engage focus audiences based on their interest and concerns.

*3. Balanced approach to the needs of both resident and refugee populations*.

With the large influx of refugees, it is critical to act and communicate in a way that recognizes and addresses the needs of both residents and refugees. While the host community has been very open to welcoming refugees and caring for them, some negative sentiment is growing that refugees might take away resources from residents and hamper access to health and other services. Balanced interventions will contribute to social cohesion and will increase trust in the government and the Ministry of Health.

## Recommended Interventions

A- Strengthening risk communications interventions:

First, coordination with non-state actors is crucial. WHO worked on facilitating the communication between CSOs and MoH through launching the health sector working group that will include representatives from local actors, UN agencies, and MoH. WHO suggests that MoH should position itself as the active steer of the health response for refugees and as a caring actor of the needs of both hosting and refugee populations.

Second, a community listening system should be established to build a regular channel of communication with both the host and the Ukrainian communities. A system for regular online updates (e.g., through a mailing list or social media group) will also be established to keep the information flow between different partners. Solidarity and unity between state actors and non-state actors will lead to better health outcomes. The partnership between health authorities and CSOs allows community voices, context, culture and concerns to be heard at the governmental level.

Third, behavioural and social insights research should be conducted to assess the perceptions of both the residents and the refugees regarding access to healthcare services, so action plans can be designed accordingly.

Finally, a deployment strategy for health information produced by the government and other non-sate actors should be prioritized.

B- Promoting healthy behaviours: through enhancing vaccination campaigns that target both the residents and the refugees noting that based on Geneva Centre of Humanitarian Studies, 167 articles were conducted from February 2022 till June 2022 tackling different health situations of those fleeing Ukraine or trapped in the country; however, none of them tackled the health promotion aspect of the RCCE pillar [12].

C- Informing about the continuity of care: communicate transparently to refugees about the opportunities they have and what services country offers. Engage health workers, CSOs, and volunteers to support these communications to decrease burden of diseases at an early stage.

D- Anticipating mental health needs through:

- Training a pool of social workers and engage health workers to detect signs of mental health impacts in refugees and colleagues.
- Building the capacity of all the social services employees to be able to detect and respond to mental health issues.

## Considerations for Actions

Considering their constituencies and area of expertise, the CSOs present have the potential and skills to be engaged in co-development of community engagement activities, including health promotion campaigns and trainings for healthcare workers and community members. However, identifying funding opportunities to sustain CSOs work is essential, especially as CSOs are running out of funds.

Some of the best practices are worth to be shared in these viewpoints. WHO was able to create a platform to link CSOs to policy makers, thus upgrading response coordination. In addition, engaging CSOs in messages testing, co-designing of interventions, monitoring and evaluation processes has been enhancing the participatory approach and leveraged the structures, systems and skills of local community organizations. RCCE interventions are only effective if they respond to the community needs and reflect its context and culture.

## Lessons learned and future implications

Lessons learned from the response in Ukraine’s surrounding countries clearly highlight that community engagement is at the core of greater acceptance and uptake of health protective measures and of social cohesion. This is largely based on trust building between authorities and communities. Although RCCE is a specific pillar in emergency preparedness and response plans [13], it is also a cross-cutting function across other pillars whose success depends on community’s support. As such, RCCE influences overall health outcomes in affected communities. RCCE readiness and preparedness are essential before the onset of any emergency, as they make sure the structures, systems and skills are there to be activated during the response phase, and can transit to recovery and building back better, in a virtuous circle. Governmental entities should strategically adopt the participatory approach to take decisions that respond to both host and refugee communities. The outcomes of these RCCE plans should contribute to better access to health information and health services, an increase in vaccination rates (routine immunization and COVID-19), better social cohesion, and enhanced mental health status among Ukrainian refugees. More research should be highlighted on the impact of such strategies on the health profile of the refugees.
